# Seasonal Stability Assessment of Reference Genes for Quantitative Real-Time Polymerase Chain Reaction Normalization in *Bombus terrestris*

**DOI:** 10.3390/cimb46020085

**Published:** 2024-02-03

**Authors:** Kathannan Sankar, Kyeong-Yong Lee, Kyu-Won Kwak, Su-Jin Lee, Young-Bo Lee

**Affiliations:** 1 Agricultural Biology Department, National Institute of Agricultural Science, Rural Development Administration, Wanju 55365, Republic of Korea; sankark774@gmail.com (K.S.);; 2 Division of Animal Diseases & Health, National Institute of Animal Science, Rural Development Administration, Wanju 55365, Republic of Korea

**Keywords:** qRT-PCR, gene expression, RefFinder, gene validation, juvenile hormone

## Abstract

Bumblebees (*B. terrestris*) play a crucial role as highly efficient biological agents in commercial pollination. Understanding the molecular mechanisms governing their adaptation to diverse seasonal environments may pave the way for effective management strategies in the future. With the burgeoning advancement in post-genetic studies focusing on *B. terrestris*, there is a critical need to normalize quantitative real-time PCR (qRT-PCR) data using suitable reference genes. To address this necessity, we employed RefFinder, a software-based tool, to assess the suitability of several candidate endogenous control genes, including actin (*ACT*), arginine kinase (*AK*), elongation factor 1 alpha (*EF1*), glyceraldehyde-3-phosphate (*GAPDH*), phospholipase (*PLA2*), and ribosomal proteins (*S18*, *S28*). These genes were evaluated for their efficacy as biological endogenous controls by examining their expression patterns across various environmental conditions corresponding to different seasons (Spring, Summer, Autumn, Winter) and tissues (ovary, fat body, thorax, head) in bumblebees. Moreover, the study investigated the significance of selecting appropriate reference genes for three key genes involved in the juvenile hormone (JH) signaling pathways: Krüppel homolog 1 (*Kr-h1*), methyl farnesoate epoxidase (*MFE*), and Vitellogenin (*Vg*). Our research identifies specific genes suitable for normalization in *B. terrestris*, thereby offering valuable insights into gene expression and functional metabolic genetics under varying seasonal conditions. This catalog of reference genes will serve as a valuable resource for future research endeavors.

## 1. Introduction

Bumblebees (*B. terrestris*) are crucial as highly efficient biological agents in the context of commercial pollination [1]. As part of a global initiative aimed at preserving endangered species and their habitats, while addressing the challenges posed by climate change on biodiversity, there is a concerted effort to sequence the genomes of all living organisms on Earth. Over the past decade, increased access to genomics data has enabled scholars to delve into a wide array of biological processes, investigating their molecular underpinnings. This access has also led to contemplation on the potential applications of this knowledge in ecology, agricultural production, and healthcare. Eusociality, the collaborative behavior seen in social insects, stands out as one of the most remarkable features in the field of science [2]. Extensive research on the theoretical foundations of eusociality has followed Hamilton’s seminal publications [3]. Genomic research has significantly contributed to addressing current challenges in understanding social evolution. A common method for determining relative gene expression is to utilize qRT-PCR with a reference gene [4]. The stability and specificity of the reference gene are crucial factors that can enhance the accuracy of gene expression measurements. The European hybrid *B. terrestris* is widely used in commercial pollination [5]. While many studies have explored the basic biology of this species, our study marks the first report on seasonal-based reference gene assessment and validation, especially in the Korean region. However, the physiological aspects underlying the transition from solitary to social behavior remain poorly understood [6]. Moreover, research on highly eusocial insects is limited in terms of its relevance due to their highly specialized nature. Bumblebees, with their relatively simple social structure, serve as exemplary subjects in this regard [7]. In temperate regions, the annual life cycle of bumblebee colonies dictates the short lifespan of queen bees. During this period, they undergo several significant physiological changes, progressing through solitary pre-hibernation, diapause, nest founding, and social and reproductive phases as mother queens [8]. Notably, hormonally regulated changes in fertility and metabolism coincide with these transitions in insects [9].

The juvenile hormone (JH) emerges as the primary regulator of insect growth and reproduction [10,11]. methyl farnesoate epoxidase (*MFE*) has been identified as a marker of JH synthesis due to its high expression in honeybee workers [12,13]. Additionally, *MFE* is found in various insect species, and in bumblebees, JH retains its fundamental gonadotropic function [10,14]. Notable indicators explored in honeybees and bumblebees include vitellogenin (*Vg*) and the gene transcription factor Kruppel homolog 1 (*Kr-h1*). *Kr-h1* is the initial regulatory transcription element responding to the JH-specific receptor in developing insects [15]. Studies have shown that *Kr-h1* expression, along with *Vg* and ovarian development in *B. terrestris* workers, relies on JH, and the presence of the queen inhibits *Kr-h1* expression [16,17]. However, it seems that while *Vg* has a greater impact on social interactions than on the workers’ reproductive state, JH signaling does not modulate *Vg* [17,18]. Development and reproduction are primarily regulated by JH, according to De kort et al. [19,20].

*B. terrestris* serves as a critical insect pollinator in Korea, with approximately 200,000 colonies cultivated annually in greenhouses to facilitate the pollination of crops such as tomatoes, strawberries, and peppers [21]. Consequently, commercially traded *B. terrestris* colonies experience exposure to various seasonal conditions throughout the year. This study aims to perform a seasonal stability assessment of seven reference genes (actin (*ACT*), arginine kinase (*AK*), elongation factor 1 alpha (*EF1*), glyceraldehyde-3-phosphate (*GAPDH*), phospholipase (*PLA2*), and ribosomal proteins (*S18*, *S28*)) for qRT-PCR normalization in *B. terrestris*. To achieve this, we conducted qRT-PCR on key genes participating in significant endocrine regulatory pathways genes (*MFE*, *Kr-h1*, and *Vg*). The assessment encompasses the diverse seasons in the Republic of Korea (Spring, Summer, Autumn, and Winter). Additionally, we confirmed target gene expression profiles in the four organs of the bumblebee: ovary, fat body, thorax, and head. Our study focuses on the stages when queens transition from solitary to seasonal phases, exploring variations in *B. terrestris* across different seasons and gene expression levels of key endocrine regulatory pathways in various tissues. Specifically, we concentrate on core genes within the juvenile hormone (JH) signaling pathway, aiming to understand the molecular determinants linked to the four seasonal conditions in fundamental JH genes through the targeted validation of reference genes. This article delves into tissue models and seasonal variations in bumblebee species.

## 2. Materials and Methods

### 2.1. Insects and Sampling Procedure

As the experimental insects for this study, *B. terrestris* colonies (European hybrid) are used as pollinators in greenhouses across different seasons in Korea: Winter (Noseong-ro beon-gill, Noseong-myeon, Nonsan-si, Chungcheonngnam-do), Summer (Chambyeol-ro, Daega-ro, Seongju-gun, Gyeongsangbuk-do), Autumn (Goheungnam-ro, Dodeok-myeon, Goheung-gun, Jeollanum-do), and Spring (Chang-ri, Yongan-myon, Iksan-si, Jeollanam-do). These colonies were maintained in the experimental pollinating form at the National Institute of Agricultural Science, RDA, Wanju, Jeonju, Republic of Korea. We maintained different colonies for rearing, and subsequently, worker bees were dispatched for commercial pollination across various agricultural sectors. The monitoring period spanned one month within greenhouses, with an additional one-week observation period outside of the greenhouse area. Worker bees were collected from three different colonies during the Spring (March 2023), Summer (June 2023), and Autumn (September 2023), based on their ages and behaviors. Winter data collection occurred in December 2022. Worker bees (ten individuals) were collected from each seasonal group of colonies. Subsequently, the collected bees were rapidly frozen in liquid nitrogen and stored at −80 °C until RNA extraction. Post-removal, each insect’s ovary, fat body, thorax, and head were examined under a stereomicroscope on sterile glass. Tissue samples were dissected and placed on crushed ice in microcentrifuge tubes with 200 μL of TRI reagent^®^ (Sigma-Aldrich, Saint Louis, MO, USA) and stored at −80 °C until RNA isolation.

### 2.2. Genes and Primer Design

One essential endocrine pathway and seven putative reference genes in *B. terrestris* were selected for investigation (Figure S1 and Table S1), along with a fundamental endocrine pathway. JH genes were employed for additional verification, and specific primer pairs were designed for amplification. All primers were designed using Primer-BLAST software (NCBI) in accordance with MIQE requirements. Primers from a previous study [22] were used for seven reference genes, and three primers for were newly designed (Table S1). After qRT-PCR validation of the generated primer pairs, the most suitable gene was selected for subsequent experiments.

### 2.3. RNA Extraction and Reverse Transcription

Total RNA from *B. terrestris* in different seasons and tissues was extracted from each biological sample using TRIzol (Invitrogen, Carlsbad, CA, USA) following the manufacturer’s instructions. The isolated RNA underwent DNase treatment using a TURBO DNAase Kit (Invitrogen, Carlsbad, CA, USA) to eliminate any DNA contamination. RNA concentrations and absorbance ratios were determined using a spectrophotometer (Sartorius StedimBiotech, Stonehouse, United Kingdom). Reverse transcription of 200 ng of absolute RNA from each seasonal and tissue sample was performed using a platinum master mix cDNA synthesis kit (AmfiRivert, GenDEPOT, Katy, TX, USA) in a total volume of 20 μL, which was subsequently diluted to 200 ng/μL and evaluated by spectrophotometric analysis. Separate master mixes were prepared for each DNA-primer combination, including “Tissues (ovary, fat body, thorax, head)” and “Spring, Summer, Autumn, Winter” mixes, which were utilized for three technical replicates distributed onto the qRT-PCR plate (9 μL per reaction well).

### 2.4. Quantitative Real-Time PCR

The RT-qPCR analysis was conducted using QuantStudio5 (Thermo Fisher Logical, made in Singapore). The SYBR Green PCR Master Mix (BiONEER, Daejeon, Republic of Korea) was employed in a triplicate reaction system following the manufacturer’s instructions. Primers were used at a concentration of 200 nM in each reaction, with a final volume of 9 μL. The PCR protocol comprised initial denaturation at 95 °C for 1 min, followed by 40 cycles of 95 °C for 15 s, 55 °C for 15 s, and 72 °C for 30 s. A final melt-curve step confirmed the absence of non-specific amplification. Cycle threshold (Ct) values of seven candidate reference genes and the target genes *MFE*, *Kr-h1*, and *Vg* were obtained at the same fluorescence threshold (0.1).

### 2.5. Selection of Reference Gene Expression Stability

RefFinder was utilized to assess the stability of potential reference genes. The delta Ct method was used based on arithmetic means and standard deviation (SD) values, and gene weights were assigned based on the rankings produced by each method [23]. The M values, representing the average expression stability, were determined for the seven candidates using geNorm across various seasons and tissue samples. Using geNorm, the normalization factor was calculated to identify the optimal number of reference genes [24]. To determine the final ranking, the geometric means of gene weights were computed [25]. NormFinder algorithms were employed to identify transcriptionally stable candidate genes by focusing on those with lower mean weights based on the arithmetically calculated mean values of stability (SV), as determined for the four seasons [26].

### 2.6. Validation of the Selected Reference Genes

The expression levels of *MFE*, *Kr-h1*, and *Vg* genes were compared across different tissue and seasoning combinations to assess the stability of the selected reference genes. Target gene expression was normalized using the comparative Ct method (2^−ΔΔCT^), both individually and in conjunction with the chosen reference gene [27]. Target gene expression was assessed using Origin Pro 2021 (Origin Lab Corporation, Northampton, CA, USA, 2021) with a one-way ANOVA test. A *p* = value of less than 0.05 indicated a statistically significant difference between samples.

### 2.7. Data Analysis

Origin Pro 2021 (Origin Lab Corporation, Northampton, CA, USA, 2021) was employed for a one-way ANOVA analysis of variance, along with the Tukey test, to determine differences in Ct values of reference genes across various seasonings and tissues. Additionally, the 2^−ΔΔCT^ method was used in Origin Pro 2021 for calculating the statistical significance level of relative transcript levels of JH pathway genes and further confirmed using the Tukey test.

## 3. Results

### 3.1. Performance of qRT-PCR Primer

Prior to qRT-PCR, examination of amplification efficiency and specificity was conducted. Agarose gel electrophoresis revealed a single band amplified by each primer set. Additionally, the qRT-PCR melting curve analysis exhibited a single peak for each reaction, confirming specificity (Figure S1 and Table S1). The PCR amplification efficiency ranged between 90.31% and 105% in our analysis.

### 3.2. Cycle Threshold (Ct) Range of Reference Genes

The RT-qPCR generated Ct values ranging from 14 to 35 across various seasonings and tissues. Lower Ct values indicated higher gene expression. *GAPDH* and *S18* consistently displayed relatively high expression levels, with Ct values ranging from 14.34 to 25.36 and 17.82 to 24.67, respectively. In both tissues and seasoning samples, *S18* and *GAPDH* emerged as the most common reference genes with the lowest Ct values, followed by *S28*, *PLA2*, *AK*, and *EF1* (Figure S2). Violin whisker plots were used to depict the expression patterns of candidate reference genes across tissues and seasonal samples.

### 3.3. Stability of Candidate Reference Genes for Seasoning

GeNorm and NormFinder analyses revealed that *AK*, *GAPDH*, *EF1*, *ACT*, and *S18* were the most stable candidate genes in seasonal samples, with *ACT* and *S18* jointly ranked as the most stable genes in these samples (Table 1 and Figure S3A–D). Moreover, *S28*, *S18*, and *ACT* emerged as stable genes for specific seasons based on NormFinder and comparative delta-Ct methods (Table 1 and Figure S4A). BestKeeper analysis indicated that S18 demonstrated the highest stability across Summer, Autumn (Table 1 and Figure S5), and all combined samples (Table S2 and Figure S7B).

### 3.4. Stability of Candidate Reference Genes for Tissue

Stability assessment of reference genes across tissue samples revealed an uneven order of stability values for the most stable genes (*S18*, *S28*, *GAPDH*, and *ACT*) across different programs. *EF1*, *PLA2*, and *AK* were identified as the least stable genes in four different tissues. Furthermore, RefFinder highlighted specific genes (*S18*, *ACT*, *AK*, and *PLA2*) as the most stable genes in different tissues, each exhibiting distinct patterns of stability (Table 2 and Figure S6E–H).

### 3.5. RefFinder Ranking of Reference Genes

The ranking of reference genes by RefFinder across all samples suggested an order: *S18* > *S28* > *EF1* > *ACT* > *PLA2* > *GAPDH* > *AK* (Table S2). The relative expression levels of JH pathway genes (*MFE*, *Kr-h1*, and *Vg*) were examined using various putative reference genes based on tissue samples and seasonal variations. The comparative Ct method (2^−ΔΔCT^) was employed for relative quantification of JH genes. The analysis demonstrated *S28* and *S18* as consistently expressed genes across all combined samples, while *EF1* exhibited consistent expression across fat body samples and all samples combined (Table S2 and Figure S7A).

### 3.6. Endogenous Control Gene Validation

**Figure 1:**
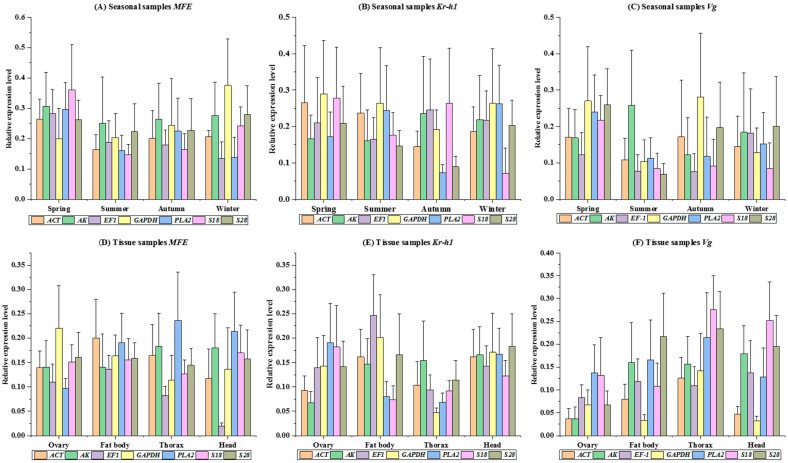
Illustration of the relative expression levels of three juvenile hormone (JH) signaling pathways, *Kr-h1*, *MFE* and *Vg*, normalized across various seasoning samples using seven reference genes. Sub-figures (**A**–**C**) represent the *MFE*, *Kr-h1* and *Vg* expression within seasonal samples, respectively. Additionally, sub-figures (**D**–**F**) display the *MFE*, *Kr-h1* and *Vg* expression within tissue samples, respectively. The values are presented as means ± SEM. Group comparisons at a significance level of 0.05 were conducted using a one-way ANOVA followed by Tukey’s multiple comparison test.

**Figure 2:**
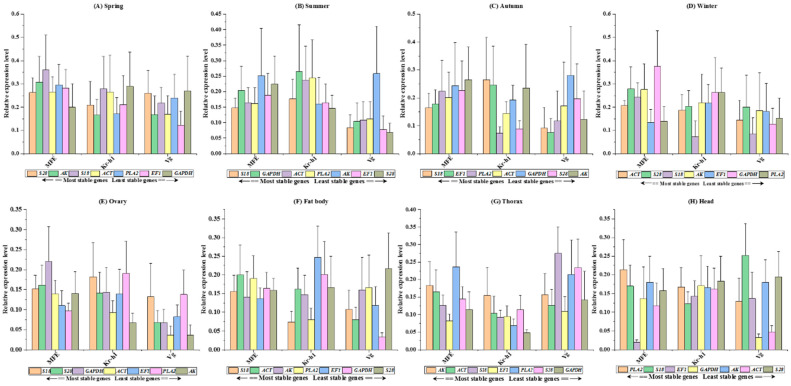
Demonstration of the validation stability of seven reference genes determined by their relative expression levels in response to three JH signaling pathways genes, *Kr-h1*, *MFE* and *Vg,* under seasoning conditions. The sub-figures (**A**–**H**) correspond to Spring, Summer, Autumn, Winter, ovary, fat body, thorax, and head samples, respectively. Presented values represent means ± SEM. Comparative analyses between groups at a significance level of 0.05 were performed using a one-way ANOVA followed by Tukey’s multiple comparison test.

**Figure 3:**
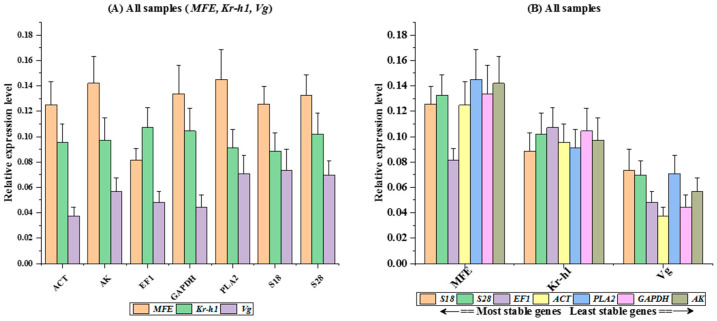
Validation of stability of seven reference genes: (**A**) determination of the expression patterns of three juvenile hormone (JH) signaling pathway genes, *Kr-h1*, *MFE* and *Vg,* under seasonal normalization using the mentioned seven reference genes. (**B**) Inducible expression levels of *MFE*, *Kr-h1* and *Vg* using *ACT*, *AK*, *EF1*, *GAPDH*, *PLA2*, *S18* and *S28* in all combined samples. Values are expressed as means ± SEM. Statistical comparisons among groups were conducted at a significance level of 0.05, employing one-way ANOVA followed by Tukey’s multiple comparison test.

The RefFinder analysis conducted in this study demonstrates the importance of utilizing multiple reference genes to normalize target gene expression. Examination of JH gene expression levels, whether normalized using individual reference genes or combinations thereof, consistently yielded results across diverse tissue types and seasonal variations (as illustrated in Figure 1A–F. This suggests that specific reference genes, namely *S18*, *GAPDH*, *PLA2*, *EF1*, or *ACT*, can effectively serve as singular normalization genes to regulate target gene expression in bumblebees across different seasonal samples (Spring: *MFE p* = 0.955, *Kr-h1 p* = 0.981, *Vg p* = 0.915; Summer: *MFE p* = 0.974, *Kr-h1 p* = 0.960, *Vg p* = 0.595; Autumn: *MFE p* = 0.993, *Kr-h1 p* = 0.751, *Vg p* = 0.903; Winter: *MFE p* = 0.503, *Kr-h1 p* = 0.559, *Vg p* = 0.991). (Figure 2A–D). Furthermore, statistical analysis of tissue-based examinations indicated that employing either a single candidate reference gene or combinations of multiple genes did not result in significant alterations in the normalized expression levels of JH genes within specific tissues. For instance, in the fat body tissue, the values were as follows: *MFE p* = 0.976, *Kr-h1 p* = 0.484, *Vg p* = 0.537; in the head: *MFE p* = 0.470, *Kr-h1 p* = 0.993, *Vg p* = 0.117; in the thorax: *MFE p* = 0.603, *Kr-h1 p* = 0.691, *Vg p* = 0.610. When considering combined tissue samples, the values were: *MFE p* = 0.238, *Kr-h1 p* = 0.980, *Vg p* = 0.152 (see Figure 2E–H and Figure 3A-B for visualization). This RefFinder analysis suggests the robustness and stability of the selected reference genes for normalizing target gene expression in various tissues and seasonal samples of bumblebees, providing valuable insights for future studies in this field.

## 4. Discussion

In Europe and Asia, the bumblebee (*B. terrestris*) is one of the most effective biological agents for commercial pollination. The concern associated with international insect transportation is that imported species could spread abroad and endanger native ecosystems. Additionally, overexploitation in the area of origin could result from uncommon species enhancing economic worth [28,29]. Since the 1990s, bumblebee colonies have been imported, significantly contributing to agricultural production. The environmental conditions or seasonal variations in Korea have a substantial impact on bumblebee colonization. Using the imported species of *B. terrestris* for greenhouse pollination offers several positive environmental impacts. These include enhanced crop yield and quality, reduced dependence on pesticides, preservation of native pollinators, conservation of wild habitats, economic benefits for local communities and minimized spread of invasive species. However, responsible management practices are essential to address potential challenges and ensure the sustainable use of *B. terrestris* in controlled environments. High-throughput sequencing methods are vital tools for investigating the genetic aspects of bumblebee attraction. These advanced techniques enable researchers to explore bumblebee genomes, analyze gene expression, and decipher the molecular mechanisms that underlie attraction behavior. This contributes to a deeper understanding of genetics and pollination biology.

Reference genes that exhibit consistent expression levels across diverse environmental conditions are crucial for obtaining more profound insights into specific gene expression patterns [30]. To date, there are no universal reference genes that are applicable to all samples and tissue types across all conditions. Therefore, before conducting a genomics study, it is imperative to evaluate the stable reference gene under various seasonal settings [31]. This study investigated seven commonly used reference genes for *B. terrestris* in the Korean region under varying seasonal conditions. The results indicate that specific reference genes (*S18*, *GAPDH*, *PLA2*, *EF1* or *ACT*) serve effectively as singular normalization genes for target gene expression in bumblebees across seasons (Spring: *MFE p* = 0.955, *Kr-h1 p* = 0.981, *Vg p* = 0.915; Summer: *MFE p* = 0.974, *Kr-h1 p* = 0.960, *Vg p* = 0.595; Autumn: *MFE p* = 0.993, *Kr-h1 p* = 0.751, *Vg p* = 0.903; Winter: *MFE p* = 0.503, *Kr-h1 p* = 0.559, *Vg p* = 0.991) (Figure 2A–D). Tissue-based analysis reveals no significant impact on normalized JH gene expression levels when using either a single candidate reference gene or combinations of multiple genes, emphasizing the stability of gene expression patterns. This underscores the importance of understanding seasonal distinctions, especially within a greenhouse setting, for the accurate interpretation of gene expression data. While many studies have validated reference genes in other insects [32], no information about bumblebee species has been published before. Consequently, this is the first publication addressing relevant reference genes in bumblebee species related to seasonal changes.

Sufficient standardization is necessary for gene expression studies in an environment that may cause errors in target gene expression [33,34,35]. The study’s findings indicate that the stability of reference genes can fluctuate based on various seasonal factors (Table 2). However, our findings demonstrated that the reference gene *S18* expression was extremely stable, routinely ranking first for both the seasonal and tissue samples. Conversely, several studies have shown that this gene is inappropriate for normalization due to their variable expression across different environmental situations (season). Analogously, studies on several insects have shown remarkable stability in *ACT* expression [36]. *ACT*, a reference gene exhibiting excellent expression stability among other genes, has been extensively utilized in insect molecular research to standardize gene expression levels. In a previous year’s instance, *S18* and *S28* in the honeybee [35] displayed the most consistent expression, showing a high degree of stability in tissues.

*B. terrestris* exhibited the greatest variation in reference gene expression among all seasons in the current study. The reference genes evaluated in the dataset of tissues sampled from the bumblebee were ranked according to stability, using five different algorithms (ovary, fat body, thorax and head). Based on the geNorm algorithms, *ACT* and *GAPDH* were the most stable reference genes. However, *EF1*, *S18* and *S28* were identified as the most stable reference genes based on the outcomes of the other four algorithms (Table 1 and Table 2). Moreover, a study on a related insect did not find such striking differences between lab-raised and wild-collected insect tissues [22]. In contrast to these geNorm methods (Table 1 and Table 2), seasonal changes significantly alter the gene expression levels in bumblebees, increasing the diversity in reference gene expression. The most stable reference genes after BestKeeper, geNorm, and delta-Ct analysis (Table 1 and Table 2) were *ACT*, *EF1*, *PLA2* and *S18.* Further evaluation of *MFE*, *Kr-h1* and *Vg* expression in various tissue samples and seasons indicated the appropriateness of the selected reference genes (Figure 2A–H). The findings suggest that, while the bumblebee was responsible for inducing *MFE* gene expression, different reference genes or gene combinations were compatible with the observed expression patterns in various seasons. However, using less-stable reference genes may lead to incorrect interpretations, as predicted by similar studies [37]. Notably, changes in expression were more pronounced during the Summer, normalized with the least stable *GAPDH* gene, than in other seasonal groups, combining the least gene *AK* and the most stable gene *ACT*, *EF1*, *PLA2* and *S18*. Furthermore, using selected single reference genes, *EF1*, *PLA2*, *S18* (the least stable) and *ACT* (the most stable), we used qRT-PCR to investigate *Kr-h1* expression patterns in different seasons (Figure 1B). More persuasive findings were observed when three genes were used for expression normalization (Figure 1A–F and Figure 2A–H). The current concept of using two or three genes to normalize target gene expression for increased accuracy was validated by these findings [38]. The most definitive finding of the evaluation was that the reference genes *S18* and *S28* were the most stable among all seasons and tissue samples examined in this investigation (Table S2 and Figure 3A). Compared to previously published studies in the field, our results indicate consistent reference gene numbers. Various seasoning effects were observed among the outcomes obtained from seasoning and tissue samples, as demonstrated by our analysis. These results imply that a single reference gene might not be the best choice for normalizing target gene expression under various seasonal circumstances. Hence, we recommend the best reference genes for each particular seasoning tissue in *B. terrestris*.

## 5. Conclusions

In conclusion, our study addresses the critical need for suitable reference genes in the context of seasonal variations in *B. terrestris*, a crucial insect pollinator in Korea. Through the comprehensive assessment of seven reference genes, including *ACT*, *AK*, *EF1*, *GAPDH*, *PLA2* and ribosomal proteins (*S18*, *S28*), across different seasons and tissues, we have identified stable reference genes that can effectively normalize quantitative real-time PCR (qRT-PCR) data. Our findings, analyzed using various algorithms such as geNorm, NormFinder, Best Keeper, and delta-Ct methods, consistently highlight *ACT*, *EF1*, *PLA2*, *S18* and *S28* as robust reference genes for seasonal normalization. Furthermore, we extend the relevance of our study by validating these reference genes in the context of key genes involved in the juvenile hormone (JH) signaling pathways, namely Kruppel homolog 1 (*Kr-h1*), methyl farnesoate epoxidae (*MFE*) and Vitellogenin (*Vg*). The stability and efficacy of these reference genes across diverse tissues (ovary, fat body, thorax and head) and seasons (Spring, Summer, Autumn and Winter) underscore their applicability in studying gene expression patterns under varying seasonal conditions. Our research contributes valuable insights into the molecular determinants associated with seasonal changes in *B. terrestris* and lays the foundation for future studies in the field. The identified reference genes, particularly *S18* and *S28*, emerge as reliable tools for researchers studying gene expression and functional metabolic genetics in bumblebees. This catalog of reference genes serves as a crucial resource for researchers in the field, facilitating accurate and standardized gene expression analyses in *B. terrestris* across different seasonal and tissue contexts. As *B. terrestris* continues to play a pivotal role in commercial pollination, understanding the molecular mechanisms underlying its adaptation to diverse seasonal environments becomes increasingly significant. Our study not only addresses this knowledge gap but also provides a practical guide for researchers engaging in molecular studies involving *B. terrestris.* Moving forward, these validated reference genes will contribute to the development of effective management strategies and further our understanding of the molecular basis of seasonal adaptations in these essential pollinators.

## Figures and Tables

**Table 1:** The expression stability of candidate reference genes across seasonal samples, including actin (*ACT*), arginine kinase (*AK*), elongation factor 1 alpha (*EF1*), glyceraldehyde-3-phosphate (*GAPDH*), phospholipase (*PLA2*), and ribosomal proteins (*S18*, *S28*), was summarized using various methods: Ct distribution analysis by geNorm, NormFinder, BestKeeper, and delta-Ct methods. The stability values were denoted by (MV) and calculated by geNorm. The standard deviation of the Ct values was analyzed by BestKeeper and delta-Ct, indicated by (SD). Additionally, the geomean ranking represents the stability values determined by RefFinder.

Rank	geNorm		Norm Finder		Best Keeper		delta-Ct		RefFinder	
	Gene	(MV)	Gene	(SV)	Gene	(SD)	Gene	(SD)	Gene	Geomean Ranking Value
Spring										
1	*AK*	0.79	*S28*	0.33	*S28*	0.29	*S28*	0.98	*S28*	1.31
2	*ACT*	0.79	*S18*	0.42	*PLA2*	0.63	*S18*	1.1	*AK*	2.71
3	*S28*	0.86	*AK*	0.78	*ACT*	0.67	*AK*	1.22	*S18*	2.82
4	*S18*	0.94	*PLA2*	0.9	*S18*	0.73	*EF1*	1.26	*ACT*	3.22
5	*EF1*	0.99	*EF1*	0.93	*EF1*	0.81	*PLA2*	1.29	*PLA2*	3.93
6	*PLA2*	1.05	*ACT*	1.01	*AK*	0.85	*ACT*	1.3	*EF1*	4.72
7	*GAPDH*	1.3	*GAPDH*	1.8	*GAPDH*	1.78	*GAPDH*	1.93	*GAPDH*	7
Summer										
1	*ACT*	0.77	*S18*	0.47	*S18*	0.32	*S18*	1.11	*S18*	1.31
2	*GAPDH*	0.77	*GAPDH*	0.76	*AK*	0.73	*GAPDH*	1.26	*GAPDH*	1.86
3	*S18*	0.91	*ACT*	0.94	*GAPDH*	0.80	*ACT*	1.35	*ACT*	1.81
4	*PLA2*	1.00	*PLA2*	1.00	*PLA2*	0.95	*PLA2*	1.41	*PLA2*	4.00
5	*AK*	1.10	*EF1*	1.05	*S28*	1.03	*EF1*	1.48	*AK*	4.35
6	*EF1*	1.22	*AK*	1.09	*EF1*	1.07	*AK*	1.49	*EF1*	5.47
7	*S28*	1.45	*S28*	1.84	*ACT*	0.14	*S28*	2.02	*S28*	6.43
Autumn										
1	*EF1*	0.66	*S18*	0.25	*S18*	0.46	*S18*	1.29	*S18*	1.00
2	*S18*	0.66	*PLA2*	0.75	*EF1*	0.71	*EF1*	1.47	*EF1*	1.86
3	*PLA2*	1.02	*EF1*	0.80	*PLA2*	0.93	*PLA2*	1.48	*PLA2*	2.71
4	*GAPDH*	1.22	*ACT*	1.13	*ACT*	0.93	*ACT*	1.64	*ACT*	4.42
5	*S28*	1.33	*GAPDH*	1.46	*S28*	0.96	*GAPDH*	1.81	*GAPDH*	4.94
6	*ACT*	1.47	*S28*	1.50	*GAPDH*	1.09	*S28*	1.84	*S28*	5.47
7	*AK*	1.67	*AK*	1.99	*AK*	1.56	*AK*	2.18	*AK*	7.00
Winter										
1	*S28*	0.78	*ACT*	0.82	*ACT*	0.45	*ACT*	1.31	*ACT*	1.00
2	*ACT*	0.78	*S28*	0.84	*S18*	0.55	*S28*	1.31	*S28*	1.86
3	*S18*	0.87	*S18*	0.92	*S28*	0.56	*S18*	1.36	*S18*	2.71
4	*AK*	1.00	*EF1*	1.03	*AK*	0.67	*EF1*	1.43	*AK*	4.47
5	*GAPDH*	1.25	*AK*	1.10	*EF1*	0.86	*AK*	1.47	*EF1*	4.68
6	*EF1*	1.36	*GAPDH*	1.16	*GAPDH*	1.09	*GAPDH*	1.55	*GAPDH*	5.73
7	*PLA2*	1.44	*PLA2*	1.38	*PLA2*	1.20	*PLA2*	1.64	*PLA2*	7.00

**Table 2:** The expression stability of candidate reference genes across tissue samples, encompassing was summarized employing diverse methods: Ct distribution analysis by geNorm, NormFinder, BestKeeper, and delta-Ct methods. The stability values were represented by (MV), calculated specifically by geNorm. For the standard deviation of the Ct values, analysis was conducted via BestKeeper and delta-Ct, denoted by (SD). Furthermore, the geomean ranking encapsulates the stability values determined by RefFinder.

Rank	geNorm		Norm Finder		Best Keeper		delta-Ct		RefFinder	
	Gene	(MV)	Gene	(SV)	Gene	(SD)	Gene	(SD)	Gene	Geomean Ranking Value
Ovary										
1	*S18*	0.58	*S28*	0.28	*S18*	0.27	*S18*	1.04	*S18*	1.18
2	*S28*	0.58	*S18*	0.29	*GAPDH*	0.41	*S28*	1.10	*S28*	1.56
3	*GAPDH*	0.68	*GAPDH*	0.30	*S28*	0.46	*GAPDH*	1.12	*GAPDH*	2.71
4	*EF1*	0.96	*ACT*	1.33	*ACT*	0.91	*ACT*	1.62	*ACT*	4.22
5	*ACT*	1.13	*EF1*	1.37	*AK*	1.08	*EF1*	1.62	*EF1*	5.14
6	*PLA2*	1.29	*PLA2*	1.4	*PLA2*	1.14	*PLA2*	1.65	*PLA2*	6.00
7	*AK*	1.40	*AK*	1.43	*EF1*	1.21	*AK*	1.67	*AK*	6.43
Fat body										
1	*S18*	0.29	*ACT*	0.14	*S18*	0.2	*S18*	0.77	*S18*	1.18
2	*ACT*	0.29	*S18*	0.14	*ACT*	0.22	*ACT*	0.78	*ACT*	1.41
3	*AK*	0.53	*AK*	0.58	*AK*	0.35	*AK*	0.89	*AK*	3.00
4	*PLA2*	0.56	*PLA2*	0.76	*PLA2*	0.45	*PLA2*	0.97	*PLA2*	4.00
5	*GAPDH*	0.67	*EF1*	0.76	*GAPDH*	0.61	*EF1*	1.07	*EF1*	5.47
6	*EF1*	0.81	*GAPDH*	0.95	*EF1*	0.7	*GAPDH*	1.12	*GAPDH*	5.47
7	*S28*	1.02	*S28*	1.47	*S28*	1.23	*S28*	1.54	*S28*	7.00
Thorax										
1	*ACT*	0.45	*ACT*	0.22	*S18*	0.23	*AK*	1.27	*AK*	1.41
2	*AK*	0.45	*AK*	0.22	*AK*	0.35	*ACT*	1.292	*ACT*	1.56
3	*S18*	0.53	*S18*	0.26	*ACT*	0.39	*S18*	1.315	*S18*	2.28
4	*EF1*	0.83	*PLA2*	1.39	*EF1*	1.14	*EF1*	1.675	*EF1*	4.22
5	*S28*	1.00	*EF1*	1.39	*PLA2*	1.14	*PLA2*	1.858	*PLA2*	4.94
6	*PLA2*	1.34	*S28*	1.90	*S28*	1.49	*S28*	2.00	*S28*	5.73
7	*GAPDH*	1.72	*GAPDH*	2.64	*GAPDH*	2.03	*GAPDH*	2.695	*GAPDH*	7.00
Head										
1	*PLA2*	0.36	*S18*	0.14	*PLA2*	0.17	*PLA2*	1.16	*PLA2*	1.18
2	*S18*	0.36	*PLA2*	0.18	*S18*	0.32	*S18*	1.25	*S18*	1.41
3	*GAPDH*	1.06	*EF1*	0.91	*EF1*	0.52	*EF1*	1.45	*EF1*	3.40
4	*ACT*	1.10	*AK*	1.35	*AK*	1.21	*GAPDH*	1.65	*GAPDH*	4.35
5	*EF1*	1.26	*GAPDH*	1.37	*S28*	1.27	*ACT*	1.73	*AK*	4.89
6	*AK*	1.38	*ACT*	1.49	*GAPDH*	1.34	*AK*	1.77	*ACT*	5.38
7	*S28*	1.59	*S28*	2.02	*ACT*	1.43	*S28*	2.13	*S28*	6.43

## Data Availability

Data are contained within the article or Supplementary Materials.

## Supplementary Materials

The following supporting information can be downloaded at: https://www.mdpi.com/article/10.3390/cimb46020085/s1, Table S1: Primer sequences and accession number of the seven candidate reference genes and target genes, Table S2: Shows the calculated expression stability values of candidate reference genes across all samples (Seasonal and tissue sample combined), as determined by gNorm, NormFinder, BestKeeper, delta-Ct, and RefFinder, Figure S1: Displays the melting curve analysis results of seven reference genes and three target genes associated with the juvenile hormone (JH) signaling pathways, Figure S2: Illustrates the distribution of Ct values’ range among the seven candidate reference genes across all four seasonal samples and four tissue samples, Figure S3: Displays the expression stability values (M) calculated by geNorm for the seven candidate reference genes across four seasonal samples, Figure S4: The figure demonstrates the Stability Ranking of Seven Reference Genes as analyzed by the ∆Ct method, Figure S5: The figure delineates the correlation existing between the Best Keeper index and the expression levels of the reference gene, Figure S6: RefFinder ranking patterns of seven reference genes in B. terrestris seasonal and tissues calculated as the geometric mean of four types of ranking (geNorm, Norm Finder, delta Ct, and Best Keeper) for each sample group, Figure S7: geNorm calculated the expression stability values (M) of the seven candidate reference genes and Comprehensive ranking patterns of seven reference genes in *B. terrestris* seasonal and tissues calculated as the geometric mean of four types of ranking (geNorm, Norm Finder, delta Ct, and Best Keeper) for each sample group.
