# Supplementary material for: Seasonal Stability Assessment of Reference Genes for Quantitative Real-Time Polymerase Chain Reaction Normalization in Bombus terrestris
Source: Curr Issues Mol Biol. 2024 Feb 3;46(2):1335–47. doi: 10.3390/cimb46020085 (PMC10887669; doi:10.3390/cimb46020085)
Supplement: Supplementary file 1 [file cimb-46-00085-s001.zip › cimb-2787246-supplementary.pdf]

Table S1. Primer sequences and accession number of the seven candidate reference genes (Actin (*ACT*), Arginine kinase (*AK*), Elongation factor 1 alpha (*EF1*), Phospholipase (*PLA2*), and Ribosomal proteins (*S18, S28*)., and three-target genes Krüppel homolog 1 (*Kr-h1*), methyl farnesoate epoxidase (*MFE*), and vitellogenin (*Vg*) in *B. terrestris*. F: forward; R: reverse; TM: Annealing temperature

Gene Symbol	Gene description	Gene bank ID	Primer sequences (5′–3′)	Product length (bp)	TM (°C)
ACT	Actin	XM_003396942.3	F "CGACTACCTCATGAAGATT"	101	56
			R "CGACAACAAAGTTTCTC"		
AK	Arginine Kinase	XM_003401454.4	F "CACACGAGGTTCACTGCTCT"	183	56
			R "GGAGAAGCCAGCTTCCAGTT"		
EF-1	Elongation factor 1 alpha	XM_012314816.3	F "GAGAAGTGCGCCGCTAGT"	94	56
			R "AACGCGAATTAAGCGGATGC"		
GAPDH	Glyceraldehyde-3-phosphate	XM_003398087.3	F"GCTGGAGCTGAATATGTTGTAGAATC"	195	56
			R "AGTAGTGCAGGAAGCATTAGAGATAACT"		
PLA2	Phospholipase A2	XM_003400908.4	F "TTGCGATGCGCATGACATTT"	114	56
			R "ATCGCAGCCGATTGATACCC"		
S18	*S18* ribosomal	XM_003400778.3	F "AGCGTGCTGGAGAATGTTCA"	101	56
			R "TCGTTCCAAGTCCTCACGAAG"		
S28	*S28* ribosomal	NW_025963548.1	F "AGCGCGGATATCTTGGACTG"	83	56
			R "TCAAGACGGGTCCTGAGAGT"		
**Target genes**					
MFE	methyl farnesoate epoxidase	KY768854.1	F"TGGATCGAAGACTACAACAG"	113	56
			R"GTGATCTGCCTACGACATTA"		
Kr-h1	Krüppel homolog 1	NW_001280921.1	F"AGTCGAGGCTGCAACTAGAAG"	179	56
			R"TTTTCTTGGAGGGAGAATTAACGT"		
Vg	vitellogenin	XM_012308109.3	F "AAGAATCATCTGAGCAACGTGA"	106	56
			R "TAGTGCACTGTTTGCTTTTGGT"		

Table S2. shows the calculated expression stability values of candidate reference genes *ACT, AK, EF1, GAPDH, PLA2*, *S18*, and *S28* across all samples (Seasonal and tissue sample combined), as determined by gNorm, NormFinder, BestKeeper, delta-Ct, and RefFinder. The stability values were represented by (MV), calculated specifically by geNorm. For the standard deviation of the Ct values, analysis was conducted via BestKeeper and delta-Ct, denoted by (SD). Furthermore, the geomean ranking encapsulates the stability values determined by RefFinder.

Rank	geNorm		Norm Finder		Best Keeper		delta-Ct		RefFinder	
	Gene	(MV)	Gene	(SV)	Gene	(SD)	Gene	(SD)	Gene	Geomean Ranking Value
1	*S28*	1.13	*S18*	0.92	*S18*	0.60	*S18*	1.67	*S18*	1.00
2	*S18*	1.13	*EF1*	1.10	*S28*	0.76	*EF1*	1.75	*S28*	2.37
3	*EF1*	1.34	*ACT*	1.20	*EF1*	0.96	*ACT*	1.85	*EF1*	2.44
4	*PLA2*	1.52	*S28*	1.38	*PLA2*	1.24	*S28*	1.90	*ACT*	3.87
5	*ACT*	1.68	*PLA2*	1.39	*ACT*	1.25	*PLA2*	1.93	*PLA2*	4.47
6	*GAPDH*	1.76	*GAPDH*	1.48	*GAPDH*	1.34	*GAPDH*	2.00	*GAPDH*	6.00
7	*AK*	1.92	*AK*	1.97	*AK*	1.53	*AK*	2.31	*AK*	7.00

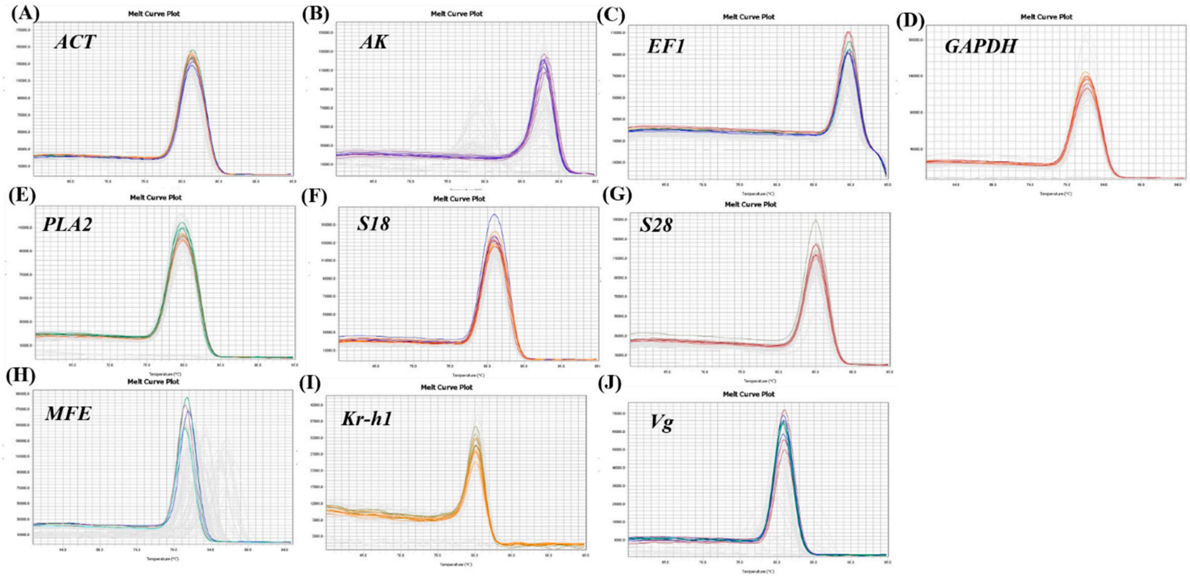

Figure S1. displays the qRT-PCR melting curve analysis results of seven reference genes and three target genes associated with the juvenile hormone (JH) signaling pathways. Individual curves, each in a distinct color, indicating varying sample replications. The reference genes include Actin (*ACT*) **(A)**, Arginine kinase (*AK*) **(B)**, Elongation factor 1 alpha (*EF1*) **(C)**, Glyceraldehyde-3-phosphate (*GAPDH*) **(D)**, Phospholipase (*PLA2*) **(E)**, and Ribosomal proteins *S18* **(F)** and *S28* **(G).** Additionally, the three JH signaling pathway target genes are Methyl farnesoate epoxidase (*MFE*) **(H)**, Kruppel homolog 1 (*Kr-h1*) **(I)** and Vitellogenin (*Vg*) **(J).**"

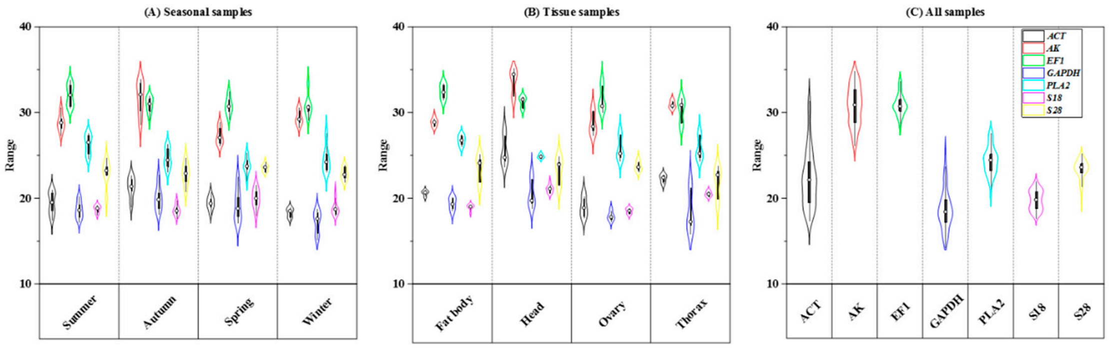

Figure S2. Illustrates the distribution of Ct values' range among the seven candidate reference genes *ACT, AK, EF1, GAPDH, PLA2, S18*, and *S28* across all four seasonal samples and four tissue samples. (**A**) Seasonal sample (**B**) Tissue samples (**C**) All samples combined; tissue and seasoning samples. Each box plot represents the median (line inside the box), the interquartile range (from the 25th to 75th percentiles - outer box), and the highest and lowest Ct values (top and bottom whiskers, respectively)."

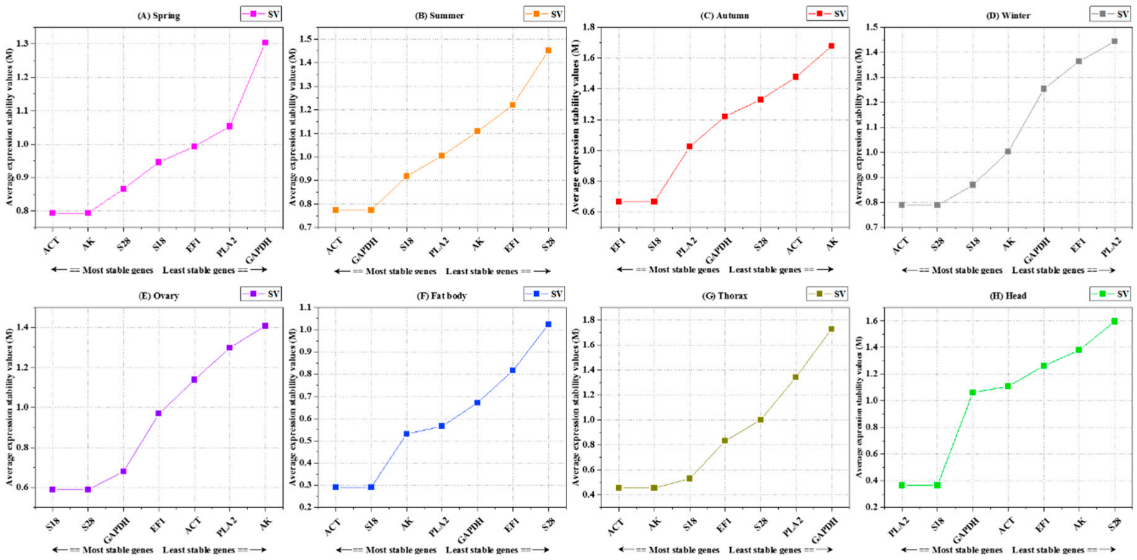

Figure S3. Displays the expression stability values (M) calculated by geNorm for the seven candidate reference genes across four seasonal samples: (A) spring, (B) summer, (C) autumn, and (D) winter, as well as four tissue samples: (E) ovary, (F) fat body, (G) thorax, and (H) head.

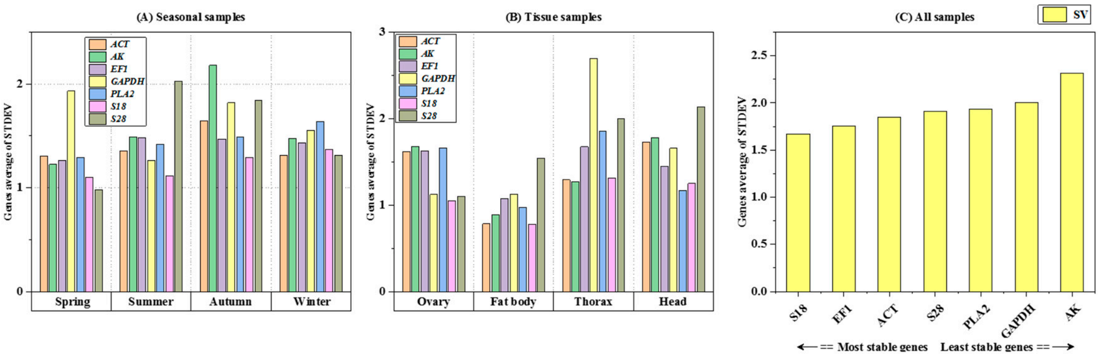

Figure S4. The figure demonstrates the Stability Ranking of Seven Reference Genes as analyzed by the ΔCt method. Sub-figure (A) displays the stability ranking across seasonal samples (spring, summer, autumn, winter). Sub-figure (B) showcases the ranking across tissue samples (ovary, fat body, thorax, head). Additionally, sub-figure (C) Illustrates the ranking across all samples, encompassing both combined tissue and seasoning samples. The data is presented as the average of standard deviation (SD).

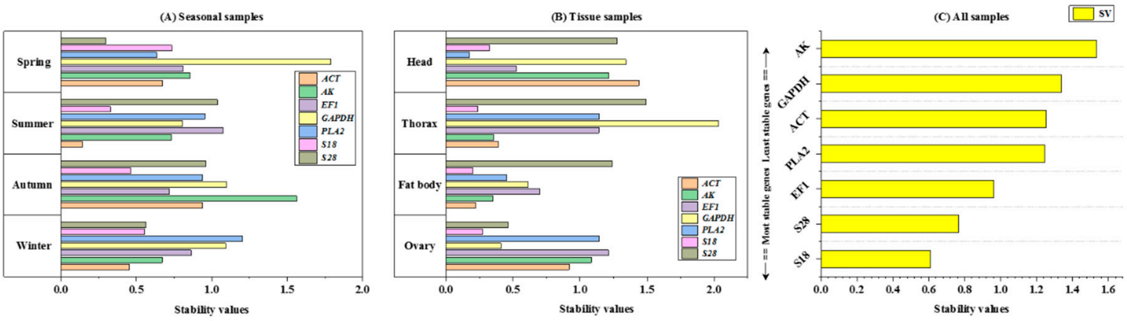

Figure S5. The figure delineates the correlation existing between the Best Keeper index and the expression levels of the reference gene *ACT, AK, EF1, GAPDH, PLA2, S18,* and *S28*. Sub-figures **(A)** and (**B**) represent the correlations within seasonal and tissue samples, respectively. Additionally, sub-figure **(C)** demonstrates the overall correlation across combined tissue and seasoning samples.

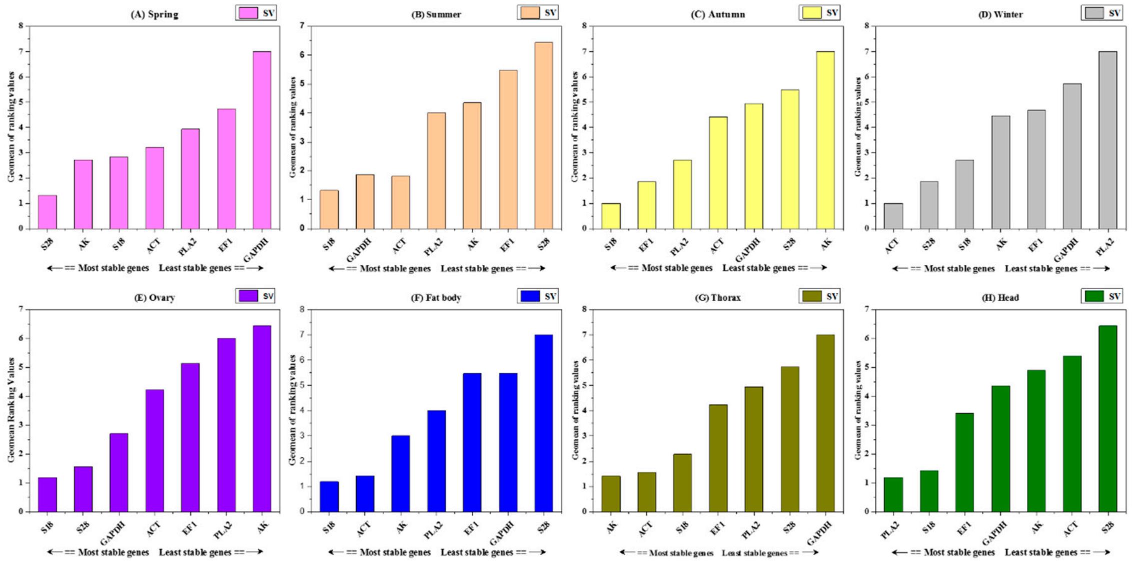

Figure S6. RefFinder ranking patterns of seven reference genes in *B. terrestris* seasonal and tissues calculated as the geometric mean of four types of ranking (geNorm, Norm Finder, delta Ct, and Best Keeper) for each sample group. The most stable reference genes for each seasonal and tissue are as follows; (A) Spring. (B) summer. (C) Autumn. (D) Winter. (E) Ovary, (F) fat body, (G) Thorax, (H) Head, " A lower Geomean value indicates more stable expression of the reference genes across different samples and tissues."

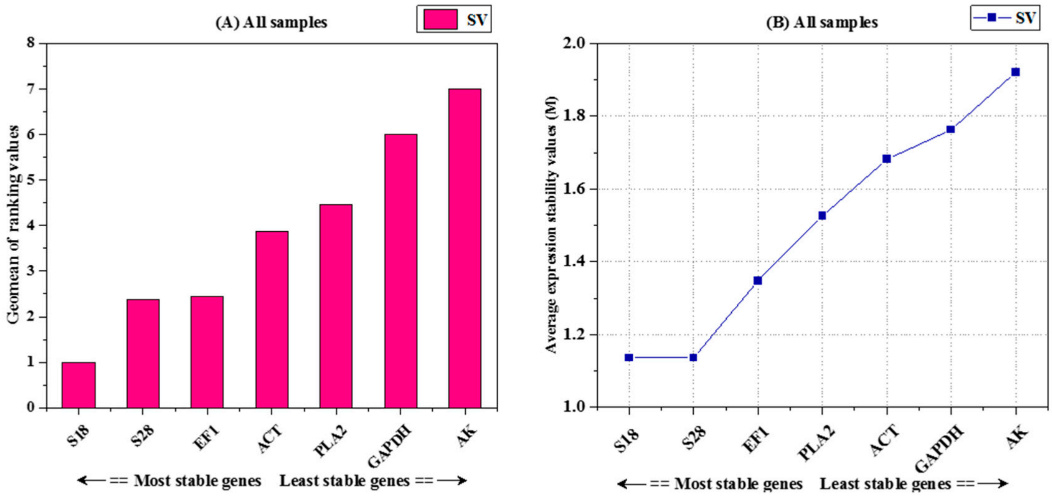

Figure S7. geNorm calculated the expression stability values (M) of the seven candidate reference genes and Comprehensive ranking patterns of seven reference genes *ACT, AK, EF1, PLA2, S18,* and *S28.* in *B. terrestris* seasonal and tissues calculated as the geometric mean of four types of ranking (geNorm, Norm Finder, delta Ct, and Best

Keeper) for each sample group. **(A)** Comprehensive ranking (All samples), **(B)** All samples (geNorm). A lower Geomean values suggests stable expression.
